# Casein phosphopeptide combined with fluoride enhances the inhibitory effect on initial adhesion of *Streptococcus mutans* to the saliva-coated hydroxyapatite disc

**DOI:** 10.1186/s12903-020-01158-8

**Published:** 2020-06-12

**Authors:** Xiaodie Wang, Limin Liu, Xiaoyan Zhou, Yongbiao Huo, Jinlong Gao, Haijing Gu

**Affiliations:** 1 Hospital of Stomatology, Guanghua School of Stomatology, Sun Yat-sen University, 56 Ling Yuan Xi Road, Guangzhou, 510055 Guangdong China; 2 Guangdong Provincial Key Laboratory of Oral Diseases, Sun Yat-sen University, 56 Ling Yuan Xi Road, Guangzhou, 510055 Guangdong China; 3 School of Dentistry, Faculty of Medicine and Health, The University of Sydney, Camperdown, NSW Australia; 4 Institute of Dental Research, Westmead Centre for Oral Health, Westmead, NSW Australia

**Keywords:** Casein phosphopeptide, Fluoride, Bacterial adhesion, Salivary pellicle, Dental caries, *Streptococci mutans*

## Abstract

**Background:**

Recent preventive strategies for dental caries focus on targeting the mechanisms underlying biofilm formation, including the inhibition of bacterial adhesion. A promising approach to prevent bacterial adhesion is to modify the composition of acquired salivary pellicle. This in vitro study investigated the effect and possible underlying mechanism of pellicle modification by casein phosphopeptide (CPP) on *Streptococcus mutans* (*S. mutans*) initial adhesion, and the impact of fluoride on the efficacy of CPP.

**Methods:**

The salivary pellicle-coated hydroxyapatite (s-HA) discs were treated with phosphate buffered saline (negative control), heat-inactivated 2.5% CPP (heat-inactivated CPP), 2.5% CPP (CPP) or 2.5% CPP supplemented with 900 ppm fluoride (CPP + F). After cultivation of *S. mutans* for 30 min and 2 h, the adherent bacteria were visualized by scanning electron microscopy (SEM) and quantitatively evaluated using the plate count method. Confocal laser scanning microscopy (CLSM) was used to evaluate the proportions of total and dead *S. mutans*. The concentrations of total, free, and bound calcium and fluoride in the CPP and fluoride-doped CPP solutions were determined. The water contact angle and zeta potential of s-HA with and without modification were measured. The data were statistically analyzed using one-way ANOVA followed by a Turkey post hoc multiple comparison test.

**Results:**

Compared to the negative control group, the amount of adherent *S. mutans* significantly reduced in the CPP and CPP + F groups, and was lowest in the CPP + F group. CLSM analysis showed that there was no statistically significant difference in the proportion of dead *S. mutans* between the four groups. Water contact angle and zeta potential of s-HA surface significantly decreased in the CPP and CPP + F groups as compared to the negative control group, and both were lowest in the CPP + F group.

**Conclusions:**

Pellicle modification by CPP inhibited *S. mutans* initial adhesion to s-HA, possibly by reducing hydrophobicity and negative charge of the s-HA surface, and incorporating fluoride into CPP further enhanced the anti-adhesion effect.

## Background

Dental caries is a biofilm-mediated multifactorial disease [1, 2]. The formation of dental biofilm is initiated by bacterial adhesion to the acquired salivary pellicle on the surface of dental hard tissues [3]. Salivary pellicle is formed immediately after toothbrushing through the selective adsorption of salivary components onto the tooth surface. The pellicle has a protective effect against dental caries by functioning as a semi-permeable barrier, and a calcium and phosphate reservoir to concurrently regulate calcium and phosphate homeostasis between tooth surface and saliva, inhibiting demineralization and promoting remineralization [3]. However, the amylase and proline-rich proteins presented in the pellicle also serve as specific binding sites for lectin-like bacterial adhesins, thereby facilitating bacterial adhesion to the pellicle-coated tooth surface [4]. The primary cariogenic bacterium *Streptococcus mutans* (*S. mutans*) has been shown to colonize on the tooth surface via the interaction with the salivary pellicle [5]. Conventional treatment of biofilm-related diseases aims at the mechanical removal of the biofilms. Recent therapeutic strategies focus on targeting the mechanisms underlying biofilm formation, including the inhibition of bacterial adhesion [6]. The alteration of molecular composition and physicochemical properties of the pellicle has been shown to disrupt the bacterial adhesion [7]. For example, modification of the pellicle by natural products, such as tannic acid and propolis, is considered to be a safe and cost-effective approach to prevent bacterial adhesion [8, 9].

Casein phosphopeptide (CPP) is milk-derived phosphorylated peptide that can be naturally present in milk or obtained by proteolytic digestion of casein [10–12]. CPP contains the cluster sequence -Ser(P)-Ser(P)-Ser(P)-Glu-Glu- which is a high polar acidic domain with high binding affinity for divalent metal ions [13]. CPP has been shown to bind and solubilize calcium efficiently, enhance the absorption of calcium in the gastrointestinal tract, improve remineralization of the tooth surface, and buffer the pH of dental biofilm [14]. Previous studies have also demonstrated the efficacy of CPP on inhibiting demineralization [15] and promoting remineralization [16] of non-carious lesion in vitro. A randomized controlled trial showed that toothpaste containing 2% CPP or 1190 mg/kg fluoride similarily reduced the incidence of caries [17]. The caries-preventive activity of CPP is attributed to its ability to stabilize high levels of amorphous calcium phosphate (ACP) on the tooth surface [18], and the CPP-amorphous calcium phosphate (CPP-ACP) complex has been developed and patented by the Reynolds group [19]. However, limited information is available on the effect of CPP on bacterial activity. 0.5% CPP solution had no significant effect on the established *S. mutans* biofilm [20]. Previous research indicated that CPP inhibited *S. mutans* and *Streptococcus sobrinus* (*S. sobrinus*) initial adhesion to the pellicle-coated hydroxyapatite surface [21]. However, the exact anti-adhesion mechanism of CPP remains unknown.

Fluoride has been recognized as an anti-caries agent over seven decades. Fluoride can inhibit demineralization and promote remineralization of dental hard tissues [22]. Fluoride also inhibits bacterial glycolytic enzyme and mediates transmembrane proton movement, which leads to bacterial cytoplasmic acidification and even kills the bacteria [23]. It has been proposed that incorporating fluoride as a phosphatase inhibitor into the CPP-containing dental care products can enhance the effectiveness of CPP during application [24]. Although several studies showed that incorporating fluoride into CPP-ACP further improves the remineralization efficacy of CPP-ACP [25, 26], there were also evidences indicating that incorporating fluoride into CPP or CPP-ACP did not improve their demineralization-suppressing effect [15, 18, 27, 28]. The impact of fluoride on the efficacy of CPP remains unclear.

The aim of this study was to investigate the effect and possible underlying mechanism of CPP on pellicle modification and *S. mutans* initial adhesion, as well as the impact of fluoride on the efficacy of CPP.

## Methods

### Casein phosphopeptide and fluoride

CPP powder (Fujifilm Wako Pure Chemical Corporation, Osaka, Japan) and sodium fluoride (Sigma, Saint Louis, MO, USA) used in this study were reagent-grade.

### Test solutions

Two-fold serially diluted CPP solutions in the range of 0.15625–10% (w/v) and 2.5% CPP solution supplemented with 900 ppm fluoride were prepared. After adjusting the pH to 7.0 using sodium hydroxide, the solutions were sterilized through a 0.22 μm polyethersulfone membrane (Merck Millipore Ltd., Tullagreen, Carrigtwohill, Co. Cork, IRL). Heat-inactivated 2.5% CPP solution was prepared by autoclaving at 121 °C for 15 min. All solutions were prepared freshly and used on the same day.

### Hydroxyapatite disc

Hydroxyapatite (HA) disc (9.7 mm in diameter and 1.5 mm in thickness, Clarkson Chromatography Products, South Williamsport, PA, USA) was used as the substrate in this study. To visualize the live and dead bacteria cells on the substrate surface, the HA disc was polished using a precision lapping/polishing machine (Unipol-1502, Kejing Auto-Instrument Co., LTD, Shenyang, China) with FEPA P #500, #1200, and #2000 silicon carbide sandpapers under constant water cooling, until the disc reached a thickness of 0.3 mm. After polishing, the disc was sonicated in ultrapure water for 10 min to remove the debris. Before experiments, the HA disc was autoclaved at 121 °C for 15 min.

### Bacterial strain and culture conditions

*S. mutans* UA159 (ATCC 700610) purchased from Guangdong Microbial Culture Collection Center was used in this study. To prepare the inoculum, *S. mutans* was firstly recovered on a brain heart infusion (BHI, Difco, Detroit, MI, USA) agar plate supplemented with 5% sterile defibrinated sheep blood for 48 h at 37 °C under anaerobic conditions (5% CO_2_, 10% H_2_, 85% N_2_). A single colony was selected and inoculated into 10 mL of BHI broth and incubated anaerobically at 37 °C overnight. Bacteria was harvested by centrifugation (3000 rpm, 4 °C, 5 min), washed twice with sterile phosphate buffered saline (PBS), and finally re-suspended in BHI broth. The optical density at 600_nm_ (OD_600nm_) was adjusted to 0.2 (corresponding to a concentration of approximately 2.0 × 10^8^ cells/mL).

### Saliva collection and preparation

Human whole unstimulated saliva was collected from eight healthy volunteers (5 females and 4 males; age range 21–27 years; mean ange 24.2 ± 2.0) recruited from students of dental faculty. The exclusion criteria include patients with systemic diseases, salivary gland disorders, active caries or periodontal disease, smoker, antibiotics or antibiotics-containing mouthwashes uses for the last 3 months before saliva collection. The aim and details of saliva collection were explained, such as not taking in anything for at least 2 h before saliva collection, and informed consent was obtained from all volunteers. The present study was approved by the Ethical Review Committee, Hospital of Stomatology, Guanghua School of Stomatology, Sun Yat-sen University (Approval No. ERC-[2017]-24). Collection and preparation of the saliva samples were performed referring to several published methods with some modification [29, 30]. Briefly, saliva collection was performed during the morning appointments, and the volunteers were asked to sit in a comfortable position and avoid swallowing or other oral movements during collection. The saliva was spat into a sterile and iced centrifuge tube before they experienced an urge to swallow the pooled saliva in the floor of the mouth. This process was repeated until 10 mL of whole saliva was obtained, which usually took about 50–60 min. The saliva from eight donors was pooled. After centrifugation at 4000 rpm and 4 °C for 20 min, the supernatant was collected and filtered using a 0.22 μm polyethersulfone membrane. Clarified saliva samples were separated into 12 mL aliquots, frozen quickly in liquid nitrogen, and stored at − 80 °C. Saliva samples were used within 6 months and thawed at room temperature prior to experiments. Saliva collection from the same donors was carried out with an interval of at least 30 days, until the present study was completed.

### Adhesive inhibitory concentration assay

Fourty-eight HA discs were individually placed into the wells of two 24-well plates (Costar, Corning, NY, USA). A volume of 1 mL of the clarified saliva was added into each well and incubated at 37 °C for 2 h. After rinsing twice with sterile PBS to obtain salivary pellicle-coated HA (s-HA) discs, the discs were randomly divided into eight groups (*n* = 6 per group): control group (PBS), and seven CPP groups containing serially diluted concentrations of CPP. The s-HA disc was incubated in 1 mL of the corresponding solutions for 2 h at 37 °C, followed by two rinses with PBS. A volume of 1 mL of the bacteria suspension was seeded and incubated for 30 min and 2 h in a humidified atmosphere of 5% CO_2_ at 37 °C (three discs per group and incubation time). After rinsing twice with PBS to remove non-attached and loosely bound bacteria, the HA disc was transferred into an eppendorf tube containing 2 mL of PBS. Adherent *S. mutans* was detached by sonication for 10 min, followed by vortexing for a further 60 s. The sonicated and vortexed *S. mutans* suspension was serially diluted and 100 μL of the diluted solution was spread over a BHI agar plate and incubated for 48 h at 37 °C in a humidified atmosphere of 5% CO_2_. The number of adherent *S. mutans* was expressed as colony-forming units (CFU) per disc. The experiment was repeated three times in triplicate. The adhesion reduction percentage was calculated as follows: (CFU counts of the control group - CFU counts of the treated group)/CFU counts of the control group × 100%.

### Bacterial adhesion assay

CPP at 2.5% (w/v) was used for further experiments based on the ability to reduce the adhesion of *S. mutans* by approximately 50% [31]. For the *S. mutans* adherence assay, s-HA disc was prepared as described above. The s-HA discs were randomly divided into four groups (*n* = 10 per group) and incubated with 1 mL of PBS (negative control), heat-inactivated 2.5% CPP (heat-inactivated CPP), 2.5% CPP (CPP) or 2.5% CPP supplemented with 900 ppm fluoride (CPP + F) for 2 h at 37 °C. Pellicle formation on HA surface and CPP adsorption onto the s-HA surface were determined by measuring the amount of proteins/peptides on the HA disc (see Additional file 1). After rinsing twice with PBS, a volume of 1 mL of the *S. mutans* suspension was seeded and incubated for 30 min and 2 h in a humidified atmosphere of 5% CO_2_ at 37 °C (five discs per group and incubation time). The number of adherent *S. mutans* was measured using the plate count method as described above and expressed as CFU per disc. The experiment was repeated three times in triplicate.

Scanning electron microscopy (SEM) was used to visualize *S. mutans* adhesion on the HA surface. Two samples from each group and incubation time were prefixed in 2.5% glutaraldehyde at room temperature for at least 3 h. After washing four to six times using ultrapure water, samples were dehydrated using gradient concentrations of ethanol (30, 50, 70, 80, 85, 90, 95, and 100%) for 15 min, and then substituted by tert butyl alcohol three times, freeze-dried, sputter-coated with gold, and examined by SEM (JSM-6330F, JEOL, Japan).

### Bacteria LIVE/DEAD staining

Adhesion of *S. mutans* was also measured using a LIVE/DEAD BackLight Bacterial Viability Kit (L7012, Thermo Scientific, USA). The polished and sterile HA discs with a thickness of 0.3 mm were used. s-HA discs were prepared and divided into four groups (*n* = 6 per group) as described above: negative control, heat-inactivated CPP, CPP, and CPP + F groups. After rinsing twice with PBS, 1 mL of the *S. mutans* suspension was seeded and incubated for 30 min and 2 h in a humidified atmosphere of 5% CO_2_ at 37 °C (three HA discs per group and incubation time). The HA disc was washed twice with 0.9% sodium chloride and then stained with 1 mL of LIVE/DEAD® BacLight™ solution at room temperature in the dark for 15 min. A volume of 1 mL of LIVE/DEAD® BacLight™ solution contained 997 μL of ultrapure water, 1.5 μL of propidium iodide (PI), and 1.5 μL of SYTO 9, which was prepared according to the manufacturer’s instructions. Dead bacterial cells with damaged membrane were finally stained in red color by PI, while live bacterial cells with intact cell membrane were stained in green color by SYTO 9. Samples were observed by a confocal laser scanning microscopy (CLSM, LSM 780, Zeiss, Oberkochen, BW, Germany) with a 20× water-immersion objective lens. Dual-channel scanning observations were performed through a green channel for SYTO 9 (excitation wavelength: 488 nm) and a red channel for PI (excitation wavelength: 543 nm). Four fields were randomly selected on each sample for scanning. Image analysis was performed with ImageJ software. The ratio of the area occupied by green or red fluorescence to the whole area on each visual field was measured. For each specimen, the ratio (%) of the area covered by total fluorescence of each color to the whole area was calculated. Subsequently, the proportion (%) of red to total fluorescence was measured. The experiment was replicated three times in triplicate.

### Ion quantified analysis

The total and free calcium and fluoride concentrations in 2.5% CPP and 2.5% CPP supplemented with 900 ppm fluoride solutions were quantified, and the corresponding bound ion concentrations were calculated. Before detecting the total ion concentrations, 1 mL of the initial solution was diluted with 19 mL of 1.0 M HNO_3_ and reacted for 24 h, followed by centrifugation at 1000 g for 15 min at room temperature; the supernatant was collected to detect the total ion concentration [32]. Before detection of free ion concentrations, macromolecular CPP with a molecular weight of approximately 3 kDa was filtered using a magnetically stirred ultrafiltration device (Amicon, Model 8200, 200 mL, Millipore) equipped with an ultrafiltration disc (1 kDa NMWL, PLAC06210, Ultracel® regenerated cellulose, Millipore). According to the instructions, 50 mL of the initial solution was added into the device; equipped with a magnetic stirrer at 300 rpm, the ultrafiltration separation was driven using high-purity N_2_ (99.999%) and the pressure was maintained within 2.4 atm. CPP was filtered and the filtrate was collected to detect the free ion concentrations. Fluoride concentration was detected by ion chromatography (IC-1100, Thermo Fisher Scientific, USA), and calcium concentration was measured using inductively coupled plasma-optical emission spectroscopy (ICP-OES, ICP-OES 730, Agilent, USA). Calcium and fluoride concentrations were expressed as mmol/L (mM).

### Water contact angle measurement

s-HA discs were prepared and divided into four groups as described above: negative control, heat-inactivated CPP, CPP, and CPP + F groups. After rinsing twice with PBS, the discs were dried under a stream of nitrogen [33]. Water contact angle was measured with a Contact Angle Meter (DMo-501, Kyowa Kogyo CO., LTD, Japan). The sessile drop technique was employed and deionized water was used as the medium (2 μL per drop). The right and left water contact angles for each droplet were measured at room temperature and averaged. For each sample, the measurement was repeated at three randomly selected regions. Finally, the result was expressed as degree (°).

### Zeta potential analysis

The electrophoretic mobilities of s-HA powder (Clarkson Chromatography Products, Williamsport, PA, USA) with and without modification were measured by a Zeta Potential Analyzer (Zetasizer Nano ZS90; Malvern Instruments, Malvern, UK) according to a published method [34]. The HA powder is the raw material for the HA disc that was used in the current study. Both the HA powder and HA disc were purchased from the same company. s-HA was prepared and divided into negative control, heat-inactivated CPP, CPP, and CPP + F groups as described above in an incubator shaker (50 rpm) at 37 °C. Five measurements of electrophoretic mobilities for each sample were averaged. The zeta potential was calculated according to the Helmholtz-Smoluchowski formula and expressed as mV.

### Statistical analysis

For all statistical analyses, SPSS v.19.0 software (IBM, Armonk, NY, USA) was used. All values were expressed as the mean ± standard deviation (SD). The inter-group differences were estimated by one-way analysis of variance (ANOVA) followed by a Turkey post hoc multiple comparison test. The level of significance was set at *p* <  0.05.

## Results

### Effect of serially diluted CPP on *S. mutans* initial adhesion

**Fig. 1:**
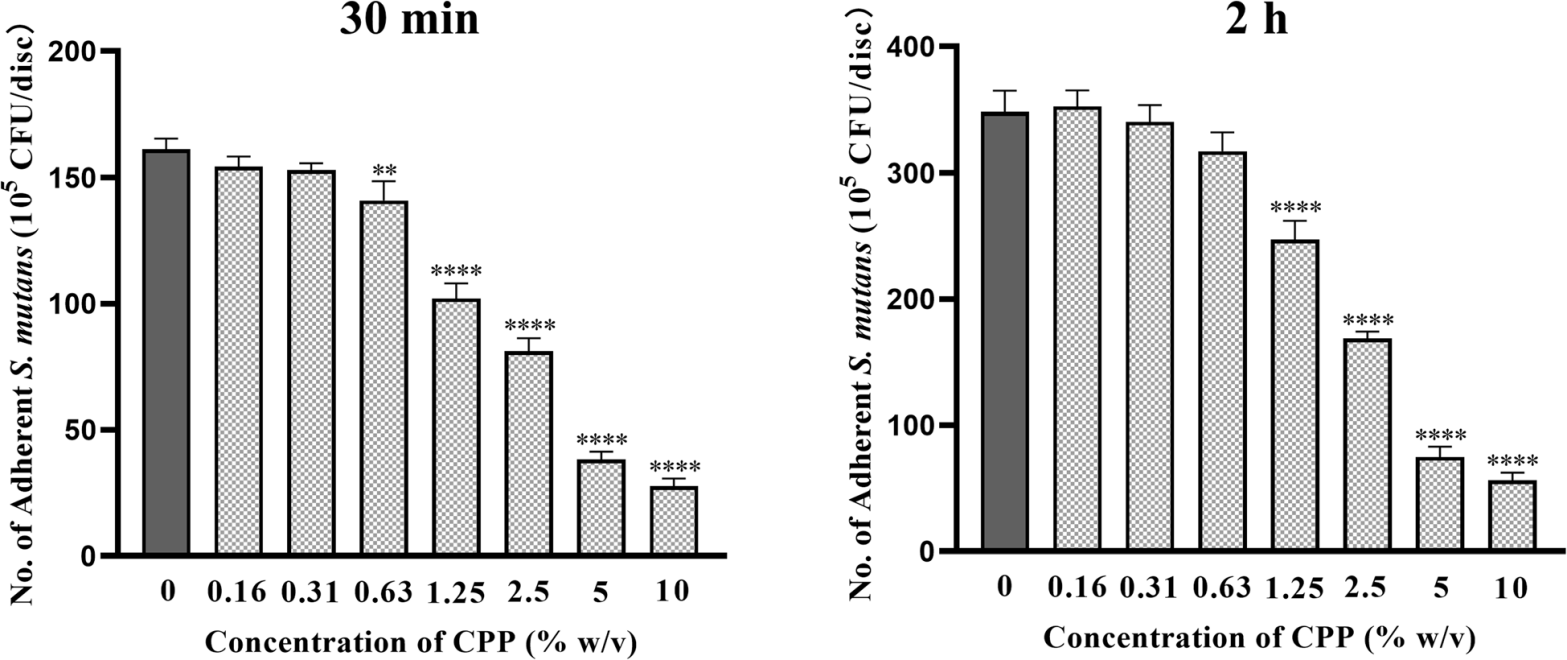
Effect of different concentrations of CPP on *S. mutans* initial adhesion. Salivary pellicle-coated HA disc was modified by two-fold serially diluted CPP solutions in the range of 0.15625–10%, followed by cultivation of *S. mutans* for 30 min and 2 h. Colony forming units (CFU) counts of *S. mutans* per disc were determined and the results were expressed as the mean ± SD. ^**^p < 0.01, ^****^p < 0.0001 compared with the control group

As shown in Fig. 1, at 30 min of incubation, compared with the control group, the reduction in the adhesion of *S. mutans* was 12.6, 36.8, 49.6, 76.2, and 82.9% for the CPP groups at concentrations of 0.63 to 10%, successively and respectively (0.63% CPP: *p* <  0.01; others: *p* <  0.0001). At 2 h of incubation, the adhesion of *S. mutans* was reduced by 32.9, 51.5, 78.5, and 83.8% for the CPP groups at concentrations of 1.25 to 10%, successively and respectively (all: *p* <  0.0001). Although the adhesion reduction in the 0.63% CPP group was 8.9% at 2 h of incubation, the difference was not significant (*p* = 0.094). There were no significant differences between the 0.16% CPP, the 0.31% CPP, and the control groups at 30 min and 2 h of incubation.

### Effect of CPP and fluoride-doped CPP on *S. mutans* initial adhesion to s-HA

**Fig. 2:**
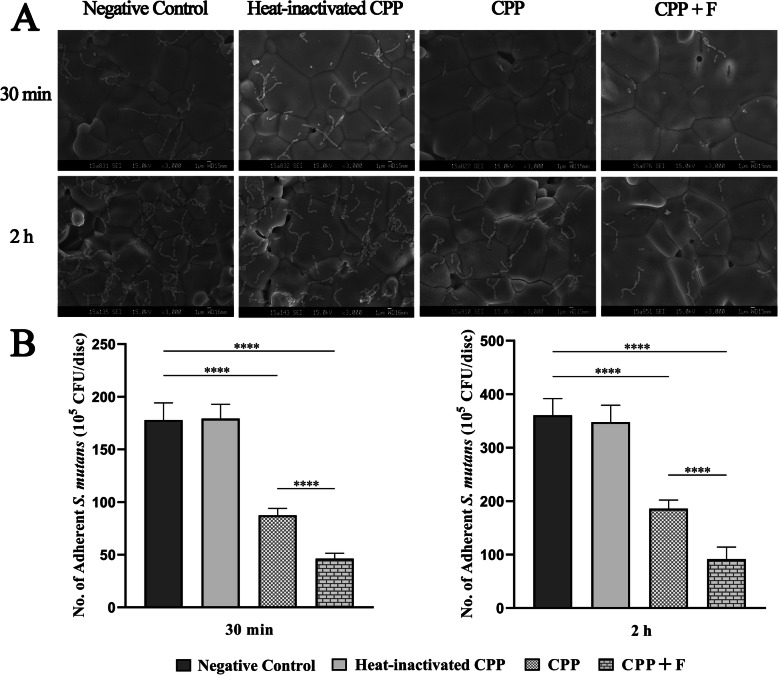
Effect of pellicle modification by CPP and fluoride-doped CPP on *S. mutans* initial adhesion. HA discs were divided into four groups: salivary pellicle-coated HA (negative control), salivary pellicle-coated HA modified by heat-inactivated 2.5% CPP (heat-inactivated CPP), or 2.5% CPP (CPP), or 2.5% CPP supplemented with 900 ppm fluoride (CPP + F). **a** Representative SEM images (3000-fold magnification) at 30 min and 2 h of incubation. **b** Colony-forming unit (CFU) counts of *S. mutans* per disc was calculated according to the plate count method. The results were expressed as the mean ± SD. ^****^p < 0.0001

The effect of 2.5% CPP and 2.5% CPP supplemented with 900 ppm fluoride on the initial adhesion of *S. mutans* to s-HA was visualized by SEM (Fig. 2a) and quantitatively evaluated by the plate count method (Fig. 2b). The formation of pellicle with CPP on the s-HA surface was confirmed by the bicinchoninic acid (BCA) method (see Additional file 1).

SEM images showed the distribution of *S. mutans* on HA discs with different coatings. The images showed a lower distribution of *S. mutans* in the CPP and CPP + F groups than that in the negative control and heat-inactivated CPP groups, both at 30 min and 2 h, and the CPP + F group showed the lowest distribution. Quantitative analysis using the plate count method showed no significant difference between the negative control and the heat-inactivated CPP groups at 30 min (*p* = 0.409) and 2 h (*p* = 0.877). At 30 min of incubation, the CFU counts were 50.8 and 74.0% lower in the CPP and CPP + F groups, respectively, than in the negative control group (both: *p* < 0.0001). The amount of *S. mutans* in the CPP + F group was reduced by 47.2% compared with that in the CPP group (*p* < 0.0001). Similar results were obtained at 2 h of incubation: the CFU counts were 48.4 and 74.5% lower in the CPP and CPP + F groups, respectively, than in the negative control group (both: *p* < 0.0001), and 50.6% lower in the CPP + F group than in the CPP group (*p* < 0.0001).

### Live and dead staining analysis

**Fig. 3:**
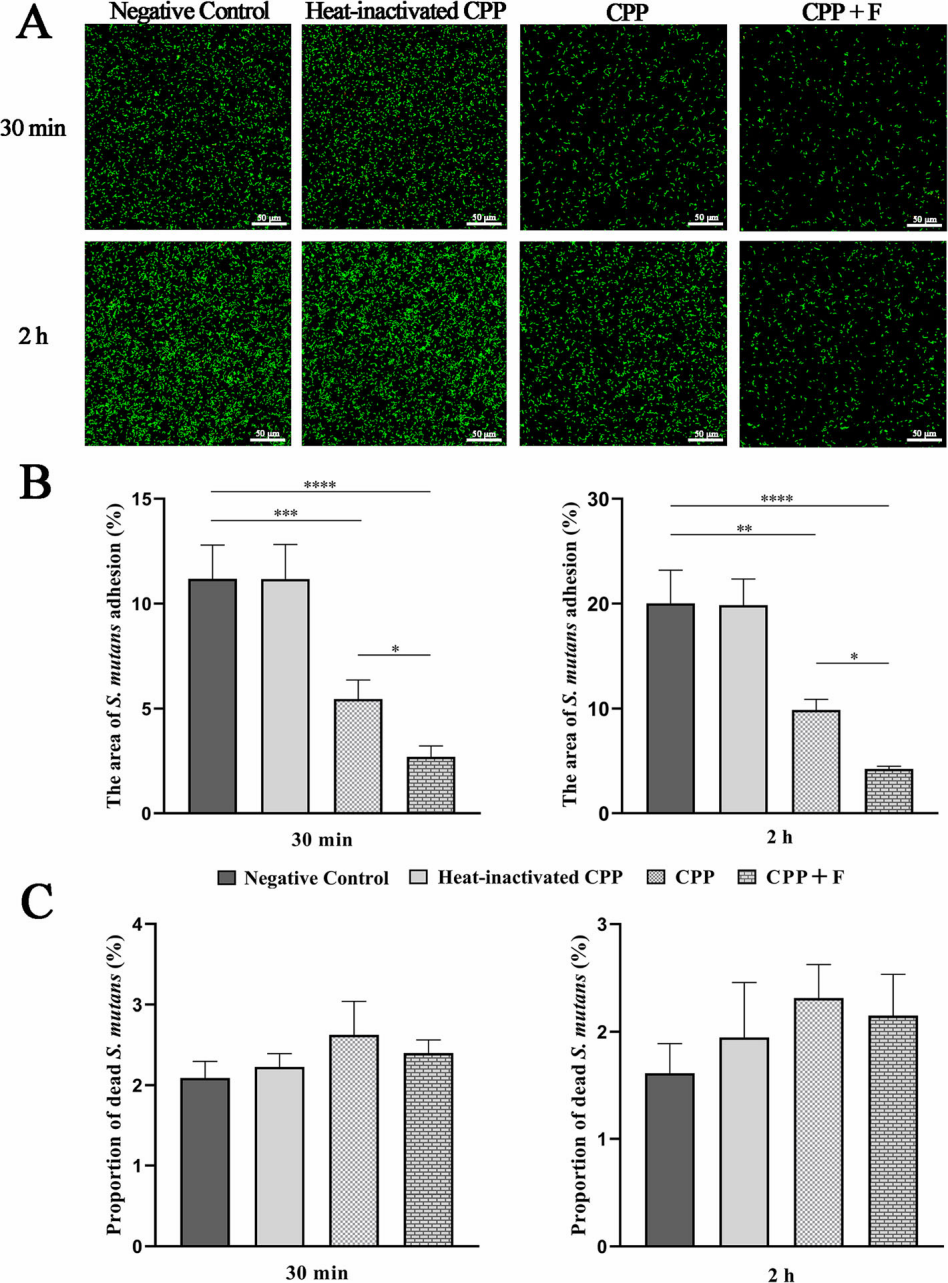
Live/dead staining analysis of *S. mutans* initial adhesion to HA discs with different coatings. HA discs were divided into four groups: salivary pellicle-coated HA (negative control), salivary pellicle-coated HA modified by heat-inactivated 2.5% CPP (heat-inactivated CPP), or 2.5% CPP (CPP), or 2.5% CPP supplemented with 900 ppm fluoride (CPP + F). **a** Representative CLSM images at 30 min and 2 h of incubation. **b** Ratio (%) of the area occupied by total fluorescence (total *S. mutans*) to the whole area. **c** Proportion (%) of red fluorescence (dead *S. mutans)* to total fluorescence (total *S. mutans*). Data were expressed as the mean ± SD. ^*^p < 0.05, ^**^p < 0.01, ^***^p < 0.001, ^****^p < 0.0001

Live and dead staining analysis was performed to confirm the anti-adhesion effect of CPP and fluoride-doped CPP, and to estimate their antimicrobial properties. As shown in Fig. 3a, fluorescence was lower in the CPP and CPP + F groups than that in the negative control and heat-inactivated CPP groups, both at 30 min and 2 h. The ratio of the area occupied by total fluorescence (total *S. mutans*) to the whole area for the negative control, heat-inactivated CPP, CPP, and CPP + F groups was 11.19, 11.17, 5.46, and 2.70% at 30 min of incubation, and 20.01, 19.84, 9.87, and 4.24% at 2 h of incubation, respectively (Fig. 3b). The area (%) occupied by *S. mutans* in the CPP and CPP + F groups was 51.23% (*p* < 0.001) and 75.84% (*p* < 0.0001) lower at 30 min and was 50.67% (*p* < 0.01) and 78.80% (*p* < 0.0001) lower at 2 h than that in the negative control group, respectively. The area occupied by *S. mutans* in the CPP + F group was 50.46% lower at 30 min and 57.01% lower at 2 h than that in the CPP group (both: *p* < 0.05). There was no significant difference between the negative control and the heat-inactivated CPP groups, both at 30 min and 2 h (both: *p* = 1).

The proportion (%) of red fluorescence (dead *S. mutans*) to total fluorescence (total *S. mutans*) was 2.09, 2.23, 2.63, and 2.40% at 30 min, and 1.61, 1.95, 2.31, and 2.15% at 2 h, for the negative control, heat-inactivated CPP, CPP, and CPP + F groups, respectively (Fig. 3c). There were no significant differences between the four groups, both at 30 min and 2 h (30 min: *p* = 0.061; 2 h: *p* = 0.213).

### Calcium and fluoride concentrations in experimental solutions

The concentrations of total, free and bound calcium and fluoride in 2.5% CPP and 2.5% CPP supplemented with 900 ppm fluoride solutions are shown in Table 1. The 2.5% CPP solution (pH 7.0) contained 30.00 mM Ca^2+^ but not F^−^ endogenously, and majority of the Ca^2+^ (29.08 mM, 96.9%) was in the bound form. The 2.5% CPP solution supplemented with 900 ppm fluoride contained 47.35 mM (899.65 ppm) F^−^, and 33.75 mM (641.25 ppm, 71.3%) F^−^ was bound by CPP, and the other was similar to the 2.5% CPP solution.

**Table 1:** Concentrations of total, free, and bound calcium and fluoride in the experimental solutions

	Ion concentration (mM)					
	Total		Free		Bound	
	Ca	F	Ca	F	Ca	F
CPP	30.00 ± 0.12	–	0.92 ± 0.03	–	29.08 (96.9%)	–
CPP + F	30.41 ± 0.04	47.35 ± 0.13	0.78 ± 0.03	13.60 ± 0.15	29.63 (97.4%)	33.75 (71.3%)

CPP: 2.5% CPP solution; CPP + F: 2.5% CPP supplemented with 900 ppm fluoride solution; Ca: Calcium; F: Fluoride. The concentrations of total and free calcium and fluoride in solutions were measured, and the corresponding bound ion concentrations were calculated. Data are expressed as the mean ± SD. Percent of ion bound by CPP is indicated in parentheses

### Water contact angle measurement

**Fig. 4:**
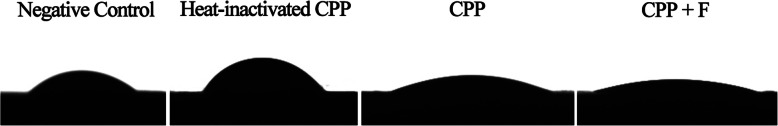
Representative images showing the contact angle of deionized water on the HA surface with different coatings: salivary pellicle-coated HA (negative control), salivary pellicle-coated HA modified by heat-inactivated 2.5% CPP (heat-inactivated CPP), or 2.5% CPP (CPP), or 2.5% CPP supplemented with 900 ppm fluoride (CPP + F)

The results of water contact angle measurement are shown in Table 2, and the representative images are shown in Fig. 4. Water contact angle significantly decreased from 42.0° (negative control) to 24.1° (CPP) and 17.4° (CPP + F) (both: *p* < 0.0001), and was smaller in the CPP + F group than in the CPP group (*p* < 0.05). The water contact angle was higher in the heat-inactivated CPP group (55.9°) than in the other groups (all: *p* < 0.0001).

**Table 2:** Water contact angle and zeta potential of the HA with different coatings

	Water contact angle (degree)	Zeta potential (mV)
Negative control	42.0 ± 1.1^a^	−9.8 ± 0.2^a^
Heat-inactivated CPP	55.9 ± 3.4^b^	−11.5 ± 0.7^b^
CPP	24.1 ± 0.8^c^	−16.4 ± 0.3^c^
CPP + F	17.4 ± 0.8^d^	−19.4 ± 0.3^d^
*p*-value	< 0.0001	< 0.0001

Negative control: salivary pellicle-coated HA; Heat-inactivated CPP, CPP and CPP + F: salivary pellicle-coated HA modified by heat-inactivated 2.5% CPP, 2.5% CPP, and 2.5% CPP supplemented with 900 ppm fluoride. Data are expressed as the mean ± SD. Within columns (water contact angle or zeta potential), different superscript letters (a, b, c, d) indicated significant difference (*p* < 0.05)

### Zeta potential analysis

All s-HA surfaces with or without modification exhibited negative zeta potential values, and the results are shown in Table 2. The zeta potential value for the negative control group was − 9.8 mV, which decreased to − 11.5, − 16.4, and − 19.4 mV for the heat-inactivated CPP, CPP, and CPP + F groups, respectively (all: *p* < 0.0001). The value for the CPP + F group was significantly lower than that for the CPP group (*p* < 0.0001).

## Discussion

A promising approach to prevent dental caries is to reduce the abundance of *S. mutans* in dental biofilm by interfering with its initial adhesion to tooth surface [33, 35]. Pellicle modification is recognized as an alternative option for improving the anti-adhesion properties of the tooth surface [36]. The present study provides evidence of the anti-adhesion effect of CPP and fluoride-doped CPP on *S. mutans*.

An in vitro model was established to simulate pellicle formation and modification as well as initial adhesion of *S. mutans*. We did not perform experiments on natural enamel, because individual enamel specimens had different chemical compositions and unclear previous history [37, 38] which may affect bacterial adhesion to enamel surface [39]. Hence, the HA disc, which is the major component of enamel, was used as the model substrate in the present study. Initial adhesion times of 30 min and 2 h were chosen to better understand the nonspecific and specific interactions between *S. mutans* and the substrate [7]. Our results demonstrated that CPP inhibited the initial adhesion of *S. mutans* to s-HA in a dose-dependent manner; a concentration of 2.5% CPP reduced the adhesion of *S. mutans* by approximately 50%, and this CPP concentration was selected for the current study [31]. The presence of endogenous calcium in the 2.5% CPP solution could be related to the fact that the CPP is produced by the proteolytic digestion of bovine casein followed with aggregation induced by calcium salt [24]. Fluoride is commonly added to various oral care products. To investigate the effect of fluoride on the efficacy of CPP, a commonly used concentration of fluoride (900 ppm) was selected for incorporation into CPP [40].

The present study indicated that initial adhesion of *S. mutans* to s-HA was inhibited after pellicle modification by CPP and fluoride-doped CPP, and the inhibitory effect of fluoride-doped CPP was most obvious. s-HA modified by heat-inactivated CPP did not appear inhibitory effect on the initial adhesion of *S. mutans*, indicating that denaturing CPP eliminated the anti-adhesion activity. The live-dead staining analysis provides evidence that the anti-adhesion effect of CPP and fluoride-doped CPP was non-bactericidal, namely *S. mutans* was prevented from attaching to the modified s-HA surface rather than being killed after adhesion. This non-bactericidal therapeutic approach targeting at bacterial adhesion can avoid the selective pressure on microorganisms which may induce resistance to treatment or proliferation of opportunistic pathogens [41].

CPP adsorption and bacterial adhesion to the pellicle are both driven by specific and non-specific interactions, such as electrostatic and hydrophobic interactions [3, 42]. To investigate the effect of pellicle modification by CPP and fluoride-doped CPP on surface properties, the zeta potential and water contact angle were measured to evaluate the charge and hydrophobicity of the s-HA surface before and after pellicle modification. Surface charge and hydrophobicity of the substrate are two important determinants that influence bacterial adhesion [43]. Decreased surface hydrophobicity is correlated with decreased bacterial adhesion [44], because initial bacterial adhesion involves the interaction between hydrophobic components on the surface of bacteria and those on the substrate [45]. Increasing the net negative charge of tooth surface reduces *Streptococcal* adhesion [46], because *Streptococcal* species (e.g., *S. mutans*) typically have a negatively charged surface [47]. The present study showed that both zeta potential and water contact angle decreased after pellicle modification by CPP. This suggested that the formation of a more hydrophilic and negatively charged surface increased the repulsion between *S. mutans* and the substrate. CPP is an amphiphiles and negatively charged peptide [24, 48]. A putative underlying mechanism mediating adsorption of CPP to pellicle is the localization of its hydrophobic sites to the pellicle surface and the orientation of hydrophilic sites toward the outer environment, thereby increasing the hydrophilicity and net negative charge of s-HA surface. This is the first study demonstrating that CPP can increase the hydrophilicity and net negative charge of s-HA surface, and this synergistic effect partially led to the reduction of *S. mutans* initial adhesion. Although the caries-preventive activity of CPP is known to be mediated by its ability to stabilize high concentrations of ACP on the tooth surface, the anti-adhesion activity of CPP demonstrated in the present study may be partially responsible for its caries-preventive effect.

Our results also showed that the anti-adhesion effect of fluoride-doped CPP was more efficient than that of CPP, attributing to the lowest water contact angle and zeta potential values found in the fluoride-doped CPP modified s-HA surface. Fluoride inhibits the adhesion of *Streptococcus sanguis* to the s-HA surface even at low concentrations [49]. The considerable amount of free fluoride (258.4 ppm) in the fluoride-doped CPP solution may play a role in inhibiting *S. mutans* adhesion by further decreasing the negative charge and hydrophobicity of the s-HA surface [40, 50]. The bound fluoride (641 ppm) may have increased the net negative charge of CPP and s-HA surface modified by fluoride-doped CPP. In general, CPP combined with fluoride enhances the anti-adhesion effect.

The present study is limited in that a single bacterial strain was investigated, and the impact of other strains was not assessed. The effects on a greater number of bacterial species should be assessed in future studies.

## Conclusions

The results of this study demonstrated that initial adhesion of *S. mutans* to s-HA can be effectively inhibited by pellicle modification by CPP, and incorporating fluoride into CPP enhances the anti-adhesion effect. We propose, for the first time, that the anti-adhesion effect of CPP and fluoride-doped CPP is non-bactericidal, and that it is mediated, at least in part, by their effect on the non-specific interactions (electrostatic and hydrophobic interactions) between *S. mutans* and s-HA.

## Supplementary information

**Additional file 1.** The optical density (OD_562nm_) of adsorbed proteins/peptides on the HA discs with different coatings. The data provided include the methods of measuring the amount of adsorbed proteins/peptides on the HA disc and the corresponding results.

## Data Availability

The datasets used and/or analyzed during the current study are available from the corresponding author on reasonable request.

## Abbreviations

CPP: Casein phosphopeptide
*S. mutans*: *Streptococcus mutans*
*S. sobrinus*: *Streptococcus sobrinus*
CPP-ACP: Casein phosphopeptide-amorphous calcium phosphate
BHI: Brain heart infusion
PBS: Phosphate buffered saline
HA: Hydroxyapatite
s-HA: Salivary pellicle-coated hydroxyapatite
CFU: Colony forming units
SEM: Scanning electron microscopy
CLSM: Confocal laser scanning microscopy
PI: Propidium iodide
SD: Standard deviation (SD)
