# Supplementary material for: Casein phosphopeptide combined with fluoride enhances the inhibitory effect on initial adhesion of Streptococcus mutans to the saliva-coated hydroxyapatite disc
Source: BMC Oral Health. 2020 Jun 12;20:169. doi: 10.1186/s12903-020-01158-8 (PMC7291725; doi:10.1186/s12903-020-01158-8)
Supplement: Supplementary file 1 — Additional file 1. The optical density (OD562nm) of adsorbed proteins/peptides on the HA discs with different coatings. The data provided include the methods of measuring the amount of adsorbed proteins/peptides on the HA disc and the corresponding results. [file 12903_2020_1158_MOESM1_ESM.pdf]

Methods

The amount of proteins/peptides adsorbed onto the HA disc before and after pellicle formation and modification was measured according to a previously described method [1]. Twelve HA discs were individually placed into the wells of 24-well plates and randomly divided into four groups (n = 3 per group): blank control, negetive control, CPP, and CPP + F groups. The native HA disc (n = 3) incubated with a volume of 1 mL of PBS was used as the blank control group. In the negetive control, CPP, and CPP + F groups, s-HA discs were performed as described in the main manuscript. After rinsing twice with sterile PBS, the s-HA dics were incubated with 1 mL of PBS (negative control), 2.5% CPP (CPP) or 2.5% CPP supplemented with 900 ppm fluoride (CPP + F) for 2 h at 37 °C. After rinsing twice with PBS, all the HA discs were transferred to a 48-well plate individually, and treated with 200 µL of micro-bicinchoninic acid (Micro BCA^{TM} Protein Assay Kit, Thermo Scientific, USA) to react with the adsorbed proteins/peptides at 37 °C for 30 min. The optical density of the supernatant at 562 nm (OD_{562nm}) was measured using a micro-plate reader (Epoch; BioTek Instruments, VT, USA). Experiments were performed three times in triplicate.

Results

Pellicle formation and modification by CPP and fluoride-doped CPP

As shown in the Supplementary Figure below, the amount of adsorbed proteins/peptides was significantly higher in the negative control (0.20 ± 0.01, $p <$ 0.05), CPP (0.77 ± 0.04, $p < 0.0001$) and CPP + F (0.77 ± 0.04, $p < 0.0001$) groups

than in the blank control group (0.15 ± 0.00). Both CPP and CPP + F groups showed significantly higher proteins/peptides amounts than the negative control group ($p < 0.0001$), whereas there was no significant difference between the CPP and CPP + F groups.

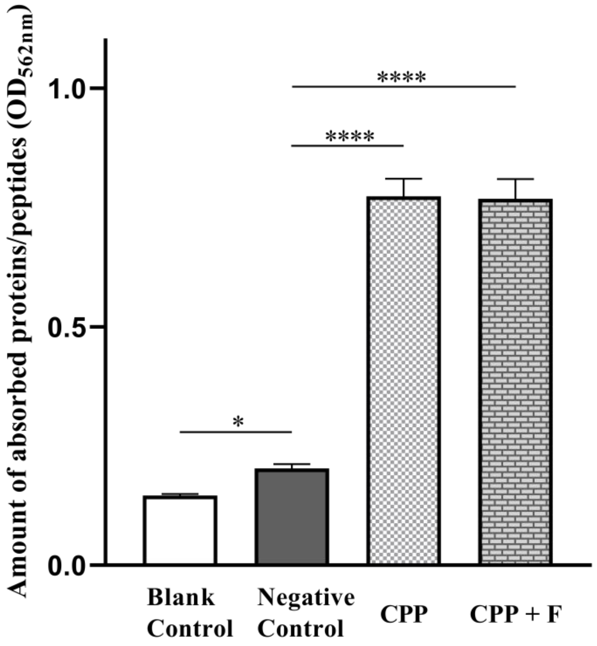

Supplementary Figure

$^{*}p < 0.05$, $^{****}p < 0.0001$.

References:

1. Kwon JS, Lee MJ, Kim JY, Kim D, Ryu JH, Jang S, Kim KM, Hwang CJ, Choi SH. Novel anti-biofouling light-curable fluoride varnish containing 2-methacryloyloxyethyl phosphorylcholine to prevent enamel demineralization. Sci Rep. 2019;9:1432.
